# An Investigation of the Magnitude of the Role of Different Plant Species in Grassland Communities on Species Diversity, China

**DOI:** 10.3390/plants13111567

**Published:** 2024-06-06

**Authors:** Miaomiao Li, Mao Ye, Yinjuan Li, Guoyan Zeng, Weilong Chen, Xiaoting Pan, Qingzhi He, Xi Zhang

**Affiliations:** 1 College of Geography Science and Tourism, Xinjiang Normal University, Urumqi 830054, China; limiao_ly@163.com (M.L.); zengguoyan@stu.xjnu.edu.cn (G.Z.); chenweilong2517@163.com (W.C.); panxiaotingcy@163.com (X.P.); he2543549455@stu.xjnu.edu.cn (Q.H.); zhangxi@stu.xjnu.edu.cn (X.Z.); 2 Xinjiang Laboratory of Lake Environment and Resources in Arid Zone, Urumqi 830054, China; 3 School of Information and Computer Science, Longdong College, Qingyang 745000, China; 18409383986@163.com

**Keywords:** dominant species, companion species, species diversity indices

## Abstract

In this study, we selected four grassland plots in Altai forest area and used the field survey method of “two-valued occurrence” to obtain the occurrence data of each plant species in the plots so as to calculate the species diversity index value of the community as a whole and the species diversity index value of each plant species not present in the community and to make use of the difference between these two diversity indices to determine the role of each plant species in the overall species diversity of the community. The difference between these two diversity indices was used to investigate the role of each plant species in the overall species diversity of the community. The results show the following: (1) In the grassland of the Altai forest area in Xinjiang, *Asteraceae*, *Poaceae*, *Fabaceae*, *Polygonaceae*, and *Rosaceae* are the dominant families, among which the genera *Puccinellia Parl*, *Taraxacum*, *Pharbitis*, *Lactuca*, *Geranium*, and *Alchemilla* are the dominant genera. (2) The plant species with the greatest contribution to species diversity in the four grassland samples was not the first dominant species of the community, but rather the plant species whose dominance was in the second to sixth position. (3) The first dominant species was overwhelmingly dominant in the four sample plots, and it served to increase the overall diversity of the community. (4) The overall trend in the size of the role of species in diversity is unimodal, i.e., logarithmically increasing to a maximum as species dominance decreases and then exponentially or linearly decreasing and eventually converging to zero. The synthesis showed that it was not the first dominant species that played the largest role in species diversity in the different grassland communities and that the overwhelmingly dominant species reduced the species diversity of the community.

## 1. Introduction

Grasslands are the most widely distributed terrestrial ecosystem on Earth, occupying more than 50% of the Earth’s land surface [1]. They have soil and water conservation functions, and in addition, they are ideal for activities such as grazing cattle and sheep [1,2]. Species diversity has always been relevant to ecosystems and has initiated an important role in the assessment of grassland ecosystem functioning. It is the basis for human survival and development and provides a contribution to sustainable development [3,4,5,6]. In addition, species diversity can also enhance grassland productivity to a certain extent and thus maintain the stability of grassland ecosystems [7,8]. Therefore, determining how to maintain species diversity in grassland ecosystems has been a hot topic of concern for many scientists at home and abroad [9,10,11,12].

Species diversity is an important dimension of biodiversity [13]. The relationship between species diversity, biodiversity, and climatic conditions has long been a hot issue in the field of ecological research [14,15,16]. On the one hand, species diversity is an important organic component of biodiversity, the most important structural and functional unit of biodiversity, and its level is key to maintaining ecosystem stability and biodiversity conservation. On the other hand, species diversity is also one of the key indicators for grassland quality evaluation [17,18]. As early as the last century, a large number of experts and scholars have done research using the species diversity index and believed that the composition of plant species plays an important role in community species diversity, such as Kruger and Taylor [19] and Zhang Xiaolong et al. [20].

Currently, the impact of livestock foraging activities on species diversity is viewed differently by different researchers. Some scientists believe that as foraging activity increases it will lead to a continued increase in surface exposure and a significant loss of soil moisture. This in turn affects the growth of water-stressed plant species and leads to a decline in the number of species [21]. It has been shown [22] that the bulk feeding of livestock leads to an increase in the bulk weight of grassland soils. However, it has also been found that overgrazing of cattle and sheep in heavily grazed areas leads to the loosening of the soil and a reduction in its bulk density [23]. National and international researchers generally agree that grassland communities have the highest species diversity during moderate foraging activities. On the one hand, the competitiveness of dominant populations with better palatability is suppressed due to selective feeding by herbivores, while those broadleaf and noxious and harmful plants that are tolerant to trampling and not palatable to livestock increase, thus increasing the species diversity of grasslands [24,25,26]. On the other hand, when herbivores feed on the more competitive plant species in the grassland community, interspecific competition is reduced. Growth and settlement conditions are provided for the weaker species as well as for the invasion of new species, which in turn increases the species diversity of the community. However, most researchers believe that the greater the degree of dominance of a plant species, the stronger the effect on community species diversity, such as MAID [27]. But what is the reality? In this study, we recorded the number of occurrences of species through field surveys, calculated their frequency, and classified the dominant species status according to the frequency from the highest to the lowest, in order to investigate how the size of the dominance of species plays a role in the diversity of species in the community.

In order to explore this issue, the present study was conducted in the Altai Forest Region. A total of four grassland sample plots were set up for field surveys in 2022 and 2020. The number of occurrences of each plant species in each site was counted using the “binary occurrence” field survey method of Shiyomi et al. [28]. On this basis, the frequency of occurrence of each plant species was calculated, and hence, the diversity index value. The role of different species in maintaining species diversity in grassland communities was explored by analyzing the size of the role of each plant species in community species diversity in different habitats. This study is expected to provide a theoretical basis for the maintenance and sustainable use of species diversity in grassland ecosystems.

## 2. Materials and Methods

### 2.1. Overview of the Study Area

The Xinjiang (China) Altai Forest Region is located in the southern foothills of the Altai Mountains, bordering Burqin County in the northwest, Jimunai County in the southwest, and Mongolia in the northeast. The terrain is high in the north and low in the south, with geographic coordinates 87°25–88°42′ E and 47°48′–48°39′ N and an altitude of 600–3914 m above sea level, belonging to a typical temperate semi-arid climate zone. The average annual temperature is around 5 °C, and the average precipitation over the years is 217.47 mm; this area is characterized by dry and hot summers and cold winters. The topography of Altai woodland is dominated by mountains. Soil types include mountain chestnut-calcium soils, mountain brown-calcium soils, and alpine meadow soils. There is a rich variety of grassland vegetation such as *Puccinellia*, *Achillea*, *Rabdosia*, *Taraxacum*, *Medicago*, *Veronica*, and *Polygonum*. Altai grassland is an important part of grassland resources in Xinjiang, so four grassland communities were selected as investigation samples along the altitudinal gradient in this study. The basic information of the sample sites is shown in Table 1.

### 2.2. Experimental Design

A survey of grassland communities in the Altai Forest Region of Xinjiang (China) was performed. The study area was divided into desert grassland, mountain grassland, mountain meadow grassland, and mountain meadow based on elevation gradient. They were used as Sample Plot 1, Sample Plot 2, Sample Plot 3, and Sample Plot 4, respectively. The number of sample plots laid out was inconsistent due to the different sizes of the sample plots, and the same survey method was used for all four plots. A representative, relatively flat section within Sample Plot 1 was selected, and a 50 m long sample line was laid. One hundred 50 cm × 50 cm sample squares were placed consecutively along the sample line, and then each sample square was evenly divided into four 25 cm × 25 cm small sample squares, totaling 400 small sample squares. A sample line of 45 m was laid out in Sample Plots 2 and 3, and 90 sample squares of 50 cm × 50 cm were placed continuously along the sample line and evenly divided into four small sample squares of 25 cm × 25 cm, totaling 360 small sample squares. In Sample Plot 4, a 60 m sample line was laid out, and 120 50 cm × 50 cm sample squares were placed consecutively along the line. Then, each sample square was evenly divided into four small sample squares of 25 cm × 25 cm, totaling 480. According to the “binary occurrence” survey method, which was first proposed and applied in frequency surveys of grassland communities by Shiyomi et al. [28], all plant species occurring in each 25 cm × 25 cm sample plot were recorded. Plant species were recorded as 1 if they appeared and 0 if they did not, and the number of times each plant species appeared in a small sample plot was counted. The frequency of occurrence of each plant species within its respective sample plots was calculated, while the list of species in each sample plot was recorded separately.

### 2.3. Dominance of Plant Species

At the beginning of the 20th century, Raunkiaer (1934) compiled a standard frequency diagram based on the frequency statistics of more than 8000 species of plants and proposed the famous Raunkiaer’s law of frequency. Species were classified into five classes, A, B, C, D, and E, bounded by a frequency of 1% to 20%, 21% to 40%, 41% to 60%, 61% to 80%, and 81% to 100% [29]. Species with frequencies in the range of E (81% to 100%) are dominant. Therefore, this study uses this as a reference to classify the dominance of species in the community.

### 2.4. Relevance Index

(1) The frequency of occurrence of a plant species, i.e., the frequency of occurrence of a species *i*, is denoted by *pi* and is calculated by the following formula [30]: *P_i_* = number of occurrences/total number of small samples in the corresponding sample plot, where the number of occurrences is the sum of the number of occurrences of the plant species in all 1 × 1 m plots in each sample.

(2) For the species diversity index for grassland communities, the Shannon–Weiner index with the following formula is utilized [31]:

(1) $$H=-\sum_{i=1}^{s}P_{i}^{’}\ln P_{i}^{’}$$

$P_{i}^{’}$ is the relative value of $P_{i}$, with the following equation:

(2) $$P_{i}^{’}=\frac{P_{i}}{\sum_{i=1}^{s}P_{i}}$$

In this formula, *S* is the number of plant species occurring in the grassland community.

(3) Each plant species in the community has a greater or lesser role in species diversity [32], but how large this role is was explored in this study using the difference in diversity indices. This difference is obtained by subtracting the value of the species diversity index of the community from the value of the diversity index of the community in the absence of a particular plant species. A positive value for the diversity index difference indicates that the presence of the species increases community species diversity, and the larger the value, the stronger the effect. Negative values indicate that the presence of the species reduces the species diversity of the community, and the larger the absolute value, the stronger the effect.

### 2.5. Data Processing

Excel 2010 software was used for preliminary data collation, standard error handling, and plotting. Correlation analyses were performed using IBMSPSS Statistics 25.0. Data on species nomenclature and ecological characters are from FRPS [33].

## 3. Results

### 3.1. Plant Frequency Characteristics of Different Grassland Types

**Figure 1:**
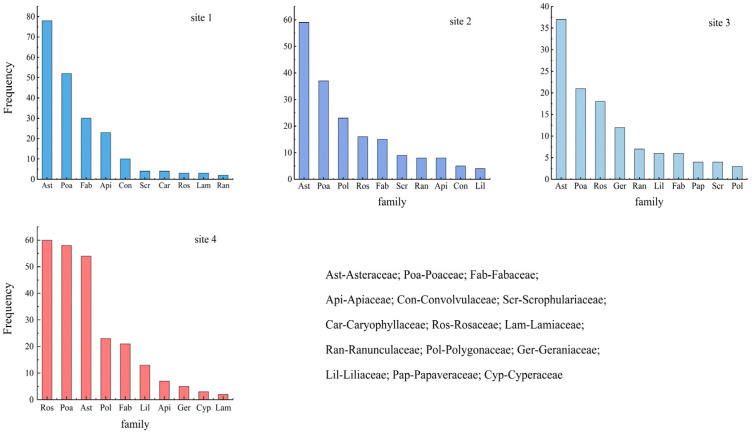
Frequency distribution of various grassland plant families (top ten).

In the investigated samples, there were large differences in the composition of plant species as well as in the dominant families among the different sites (Figure 1). In Sample Plot 1, *Asteraceae*, *Poaceae*, and *Fabaceae* were the main dominant families. The *Asteraceae* frequency was 78, accounting for 36.1% of the total frequency, which was absolutely dominant. *Poaceae* accounted for 24.1% of the total and showed sub-dominant characteristics, while *Fabaceae* accounted for 13.9% of the total. In Sample Plot 2, *Poaceae*, *Asteraceae*, and *Polygonaceae* were the main dominant families. The proportion of *Polygonaceae* increased from 0.4% in site 1 to 11.9% in site 2. The proportion of *Rosaceae* also increased considerably, while the proportion of *Asteraceae* decreased from 36.1% in site 1 to 19.1% in site 2. *Poaceae* increased from 24.1% in site 1 to 30.4% in site 2. In Sample Plot 3, *Asteraceae*, *Poaceae*, and *Rosaceae* were the main dominant families, accounting for 29.15%, 16.5%, and 14.2%, respectively. The *Geraniaceae* family appeared in the grassland community with increasing elevation and accounted for 9.4% in Sample Plot 3. *Fabaceae* and *Polygonaceae* are gradually degraded in this sample plot, with a frequency of 4.7% and 2.4%, respectively. In Sample Plot 4, *Rosaceae*, *Poaceae*, and *Asteraceae* were the main dominant families, accounting for 24.4%, 23.6%, and 22.0%, respectively.

**Figure 2:**
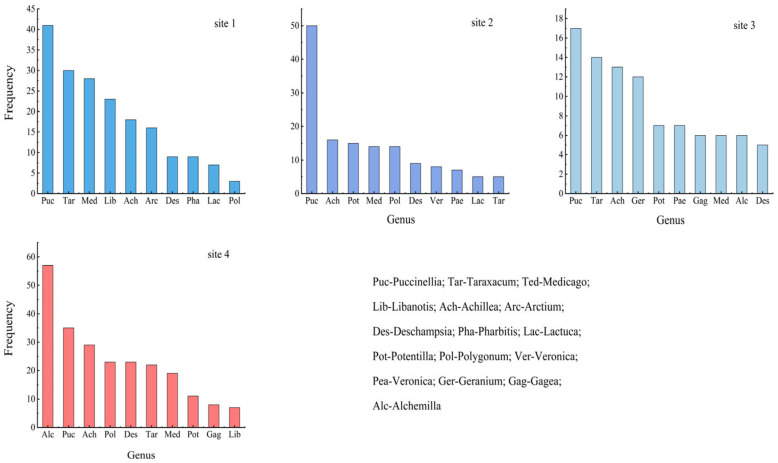
Frequency statistics of plant genera in different grassland types (in the top ten).

As can be seen from Figure 2, *Puccinellia* was the main maintainer of grassland ecological functions in all the samples, but the proportion varied more markedly among the different grassland samples. It was the most dominant genus in all the sample plots, except in Sample 4 where it represented only 13.6% and in Sample 2 where it represented 28.4%. In Sample Plot 1, *Taraxacum* and *Medicago* showed subdominant characteristics with 13.8% and 12.9%, respectively. At the same time, *Libanotis*, *Achillea*, *Arctium*, *Deschampsia,* and other genera are present. In Sample Plot 2, the genera *Pharbitis* and *Lactuca* disappeared with the increase in altitude, and the genera *Potentilla* and *Polygonum* appeared with a high percentage of 8.5% and 8.0%, respectively. In Sample Plot 3, the genera *Geranium* and *Alchemilla* appeared with the increase in altitude, with the proportion of 98% and 4.9%, respectively, and *Polygonum* disappeared. In Sample Site 4, the proportion of *Puccinellia* fescue declined and was no longer the most dominant genus, while *Alchemilla* showed the most dominant feature, accounting for 22.2% of the total. *Deschampsia* was the second most dominant genus, accounting for 8.9% of the total.

### 3.2. Characteristics of the Number of Species in Different Grassland Communities

The total number of species in each sample site was obtained as 35, 31, 22, and 22 by field survey of 25 cm × 25 cm small samples in four sample sites, respectively. Table 2 lists the top six plant species in each sample site in order of magnitude of occurrence. The table shows that these six plant species have included the dominant and major companion species in the community. It can also be seen from the table that the number of occurrences and frequency of plant species comprising the community gradually decreased as the dominance of the plant species decreased in Sample Plot 1, Sample Plot 2, Sample Plot 3, and Sample Plot 4. In contrast, the respective first dominant species of *Puccinelli adistans* in Sample Plot 1, Sample Plot 2, and Sample Plot 3 were overwhelmingly dominant in the community, and there was a large difference in the number of occurrences and frequency of their occurrences compared to that of the species whose dominance was in the second position.

### 3.3. The Role of the Various Plant Species That Make up the Community in Species Diversity

**Figure 3:**
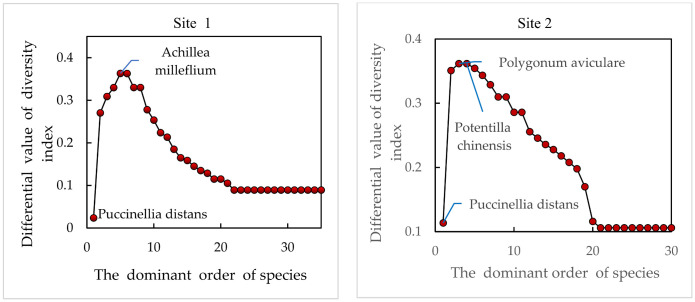

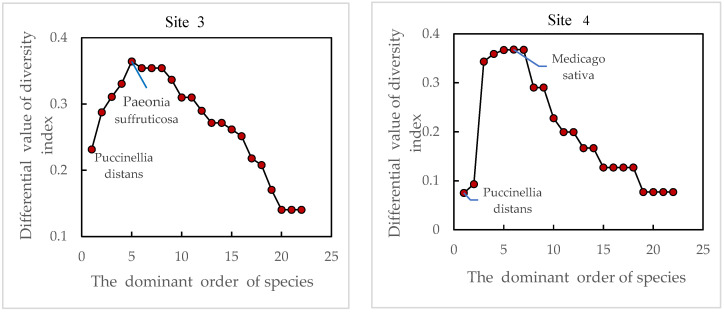
Role of each plant species in species diversity in different habitats.

Figure 3 represents the magnitude of the role of each plant species in the diversity of community species in each of the sampled grassland communities. The difference in diversity indices of the vertical coordinates of the graph represents the difference between the diversity index value of the grassland community as a whole and the diversity index value of the community in the absence of each of the plant species that make up the community. Each point represents the size of a plant’s role in community species diversity. Overall, the difference in diversity indices increased rapidly as the dominance of the plant species comprising the community decreased, then decreased rapidly after reaching the peak, and finally reached a stable state close to 0. The differences in the diversity indices of the plant species comprising the community increased rapidly as the dominance of the plant species comprising the community decreased. That is, the role of plant species in the overall species diversity of the community first increases and then decreases as their dominance decreases, and finally, the species has almost no role.

As seen in Figure 3, the difference in diversity indices of the four sample plots was positive; i.e., all plant species comprising these four communities contributed to the increase in diversity of the communities. In Sample Site 1, the plant *Achillea milleflium*, which was in the fifth position of dominance, contributed the most to the species diversity of this community, while the first dominant species, *Puccinelli adistans*, contributed less to the species diversity of the community. In Sample Site 2, the plant species *Polygonum aviculare* and *Potentilla chinensis*, which were in the third and fourth positions of dominance, contributed the most to the diversity, while the plant *Puccinelli adistans*, which was in the first position in the order of dominance, contributed relatively little to the diversity of the community species. In Sample Site 3, the plant *Paeonia suffruticosa*, which was in the fifth position of dominance, contributed the most to diversity, while the plant *Puccinelli adistans*, which was in the first position, did not contribute much to diversity. In Sample Plot 4, the plant *Medicago sativa* in the sixth position of dominance had the greatest contribution to diversity, while the plant *Puccinelli adistans* in the first position had a lesser contribution to diversity.

An overall analysis of Figure 3 shows that most of the plant species that make up the community contribute to the increase in species diversity. The effect of species on diversity in the four sample plots followed the same trend and was of the single-peaked type. There is an “inflection point”, and the frequency of plant species at the “inflection point” is between 30% and 45% (Table 2), which is indicative of not a dominant species but a companion species. This suggests that it is not the dominant species that plays the greatest role in community species diversity, but rather the companion species at “inflection points”. Therefore, in this study, the species were divided into two parts using the “inflection point” as a boundary, with the species at and before the “inflection point” as the dominant species and the main companion species. Species after the “inflection point” are used as other companion and occasional species to further explore the role of different plant species on community species diversity. And based on this, we further explore the magnitude of the role of different plant species on community species diversity.

### 3.4. The Role of Dominant and Key Associated Species in Species Diversity

**Figure 4:**
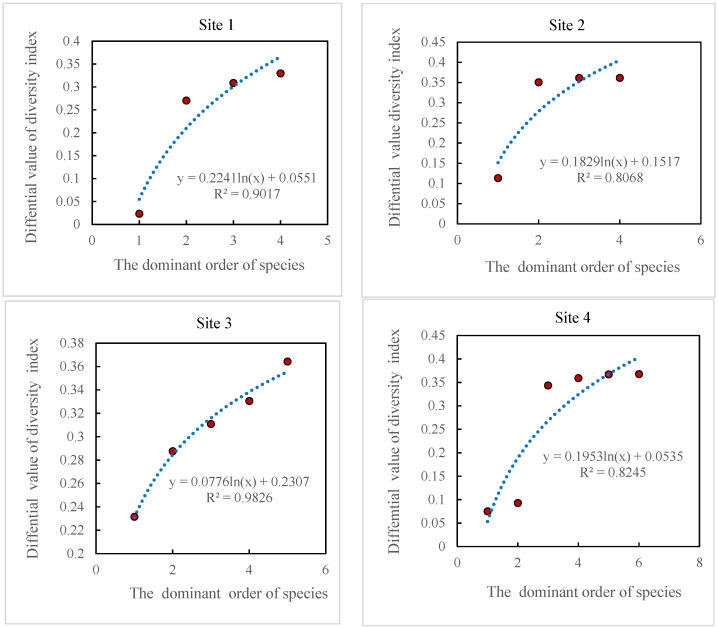
Role of dominant and key associated species in species diversity.

As can be seen in Figure 4, the size of the role of species in diversity showed a logarithmic upward trend as the order of dominance of the dominant and main companion species decreased in the four sample sites, and the degree of the role was significant (*R^2^*: 8068~0.9826), which suggests that the role of the plant species that make up the community from dominant to companion species in species diversity is an increasing process.

### 3.5. Size of the Contribution of Other Companion and Occasional Species to Species Diversity

**Figure 5:**
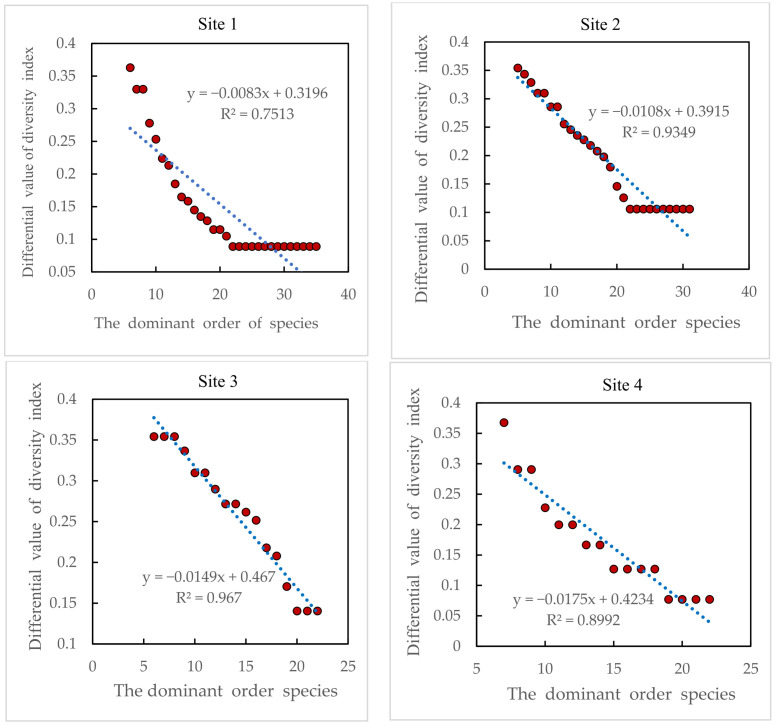
Role of other companion and occasional species in species diversity.

As can be seen in Figure 5, all species contributed to the increase in community species diversity with a positive diversity index difference. However, as the order of dominance of these companion and occasional species declined, the effect of plant species on species diversity showed a highly significant exponential or linear downward trend (*R^2^*: 0.891~0.967). That is, these companion and occasional species that make up the community have a highly significant reduced role in species diversity as their dominance decreases. Eventually, approaching 0, it plays little or no role in community species diversity.

## 4. Discussion

### 4.1. Characteristics of Grassland Plant Species in Altai

Grasslands in Xinjiang show significant vertical zonation characteristics, which makes the climatic factors show a wider range of variations on a smaller spatial scale, thus giving rise to a diversity of grassland types. Studies in the Qilian Mountains found that desert grasslands are dominated mainly by *Asteraceae* and *Poaceae* [34], which is similar to the results of this study. The families and genera of grasslands in Altai showed a trend of increasing and then decreasing, and the species showed a trend of decreasing and then increasing and then decreasing. This is somewhat different from the study in Burqin [35] where the families, species, and genera of grasses all showed an increasing, then decreasing, then increasing trend. The main reason is that in the spatial distribution pattern of grassland species, the environment of the study area is characterized by territoriality, so the pattern of species change is not the same. Therefore, differences in the dominant families may be due to different scales of observation or to anthropogenic factors such as grazing. Therefore, forms of ecological protection such as grazing bans should be implemented for the grassland with a single composition of family, genus, and species, and the grassland should be reasonably utilized according to local conditions, so as to jointly promote the healthy and sustainable development of the grassland in Altai forest area.

### 4.2. Determination of Plant Species Dominance in the Community

The grassland area in Xinjiang accounts for 34.45% of the total land area of the whole territory and is one of the important grassland resources [17,36]. At present, the Altai forest area suffers from grassland degradation, desertification, and grass–livestock imbalance. Therefore, it is important to explore the magnitude of the influence of plant species composition on species diversity in grassland communities in the Altai Forest Region. In this study, four grassland types, i.e., four sample plots, were selected as typical grassland communities along the altitudinal gradient in 2020 and 2022 in the Altai Forest Region. Data were collected in each sample plot and in all sub-samples within the plot through the “binary occurrence” field survey method. The frequency of occurrence of plant species was used to determine their order of dominance in the community, with reference to the well-known Raunkiaer’s law of frequency from the early 20th century [29]. Although the most commonly used methods regarding the classification of dominant species are the combined dominance ratio of Japanese scholar Sumida Shin (1957) and the classical importance value formula of Curtis (1951), methods such as combined dominance ratios and importance values need to be calculated using at least three of these metrics such as height, frequency, density, cover, and biomass of species in the community [37]. One of the biomass metrics requires mowing, which can have a damaging effect on the grass, whereas only the frequency metric, which does not have a damaging effect on the grass, was used in this study. In conjunction with Raunkiaer’s law of frequency, a large amount of frequency survey data was used. And because the frequency of occurrence of species in grassland communities shows a positive correlation with density, cover, and biomass [38], frequency data alone were used in this study to determine the dominance of species in the community.

### 4.3. Size of the Role of Different Species in Community Species Diversity

Species diversity index values were calculated for the community as a whole, as well as the diversity index values in the absence of each of the plant species that make up the community, and the difference between these two values was used to explore the role of each plant species in the overall diversity of the community. The results show that as the dominance of plant species of the constituent communities decreases, their role in species diversity tends to increase and then decrease. This indicates that there is a tendency for species to increase and then decrease their contribution to the overall diversity of the community as the order of plant species dominance decreases in the community. Species at “inflection points” contribute most to diversity, and these plant species play an important role in increasing species diversity. However, they are not dominant species, but major companion species with lower dominance. This is an important finding of this study, which is different from most of the scholars, such as GUO et al. [39] who found that species diversity decreases with increasing abundance of dominant species.

This study also found that both companion and occasional species affect the species diversity of the community to a greater or lesser extent. And the diversity index difference was generally positive; i.e., these species can increase species diversity, which is consistent with the findings of most scholars [40,41]. However, this finding differs from the study of Wang Lijun [42] and others, who concluded that when the plant species with the dominance order of No. 1 is absolutely dominant in the community, it will reduce the overall species diversity of the community. That is, the presence of this species will instead lead to a decrease in species diversity. The reason may be that the reduction in the first dominant species reduces the interspecific competition in the community. This allows other plant species to be fueled, increasing the species diversity of the community and resulting in a smaller difference in the diversity index. In this study, when the plant species with the dominance order of first position was absolutely dominant in the community, it did not make the community as a whole less species-like. The reason may be that its reduction increases interspecific competition in the community, making other plant species inhibited in drawing nutrients. And it is an impediment to invasive alien species to the extent that it does not contribute as much to the overall diversity of the species as the main companion species.

Studies analyzing other companion and occasional species have found that the role of these plant species in diversity tends to decrease exponentially or linearly as dominance declines until a steady state is reached. That is to say, their role in species diversity becomes smaller and smaller and finally nearly disappears. This is consistent with the findings of Waheed [43].

In view of the above analyses, it is considered that in the conservation and use of grassland communities, in order to maintain higher species diversity, it is necessary to pay attention not only to the conservation of dominant species, but also to the role of companion species in the community to achieve sustainable developmental use of grassland resources.

## 5. Conclusions

In this study, the family, genus, and species composition of the species in each sample site were counted. The composition of grassland species in the Altai forest area of Xinjiang is mainly based on *Asteraceae*, *Poaceae*, *Fabaceae*, *Polygonaceae*, and *Rosaceae* as the dominant families and *Puccinellia Parl*, *Taraxacum, Pharbitis*, *Lactuca*, *Geranium*, and *Alchemilla* as the dominant genera. However, the proportion of each family and genus in different types of grassland is different. Analyses using Raunkiaer’s law of frequency revealed that the plant species in different grassland communities that played the greatest role in the overall species diversity of the community was not the dominant species in that community, but rather a companion species. Whereas some plant species are absolutely dominant in grassland communities, they are not the ones that contribute the most to the species diversity of grassland communities. It was also found that the size of the role of species in diversity showed a unimodal trend as the order of dominance of plant species changed across grassland communities. This suggests that as the dominant species and the dominant companion species decrease in dominance order, their role in species diversity first reaches a peak, then decreases as the dominance of other companion and occasional species decreases, and finally reaches a stable value or finally has almost no effect on the overall species diversity of the community.

## Figures and Tables

**Table 1:** Basic information of the sample site.

Site	Position	Longitude and Latitude	Altitude (m)	Investigation Date
1	Desert Steppe	88°07′53.53″ E	1119.881	2022.07
		47°57′26.13″ N		
2	Montane steppe	88°15′32.35″ E	1418.409	
		47°59′43.57″ N		
3	Montane meadow steppe	87°36′09.62″ E	1714.286	2020.09
		48°09′31.92″ N		
4	Mountain meadows	88°21′26.83″ E	1973.333	
		48°00′29.54″ N		

**Table 2:** Basic information on the main species ranked in order of dominance.

Site	Dominance Order	Species Name	Occurrence	Frequency
1	1	*Puccinellia distans*	394	0.976
	2	*Taraxacum mongolicum*	348	0.667
	3	*Medicago sativa*	338	0.595
	4	*Typha latifolia*	240	0.548
	5	*Achillea milleflium*	208	0.429
	6	*Arctium tomentosum*	168	0.310
2	1	*Puccinelli adistans*	320	0.878
	2	*Achillea milleflium*	210	0.485
	3	*Potentilla chinensis*	120	0.333
	4	*Polygonuma viculare*	108	0.303
	5	*Medicago sativa*	99	0.272
	6	*Veronica didyma*	88	0.242
3	1	*Puccinellia distans*	275	0.762
	2	*Taraxacum mongolicum*	239	0.667
	3	*Achillea milleflium*	223	0.620
	4	*Gernium wilfordii*	205	0.571
	5	*Paeonia suffruticosa*	120	0.333
	6	*Alchemilla japonica*	102	0.286
4	1	*Puccinelli adistans*	444	0.921
	2	*Alchemilla japonica*	433	0.902
	3	*Achillea millefolium*	243	0.509
	4	*Taraxacum mongolicum*	216	0.451
	5	*Deschampsia caespitosa*	187	0.392
	6	*Polygonum aviculare*	180	0.372

## Data Availability

The data that support the findings of this study are available from the corresponding author upon reasonable request.
